# Interaction between extracellular lipase LipA and the polysaccharide alginate of *Pseudomonas aeruginosa*

**DOI:** 10.1186/1471-2180-13-159

**Published:** 2013-07-13

**Authors:** Petra Tielen, Hubert Kuhn, Frank Rosenau, Karl-Erich Jaeger, Hans-Curt Flemming, Jost Wingender

**Affiliations:** 1 Department of Aquatic Microbiology, University of Duisburg-Essen, Faculty of Chemistry, Biofilm Centre, Universitätsstrasse 5, D-45141, Essen, Germany; 2 University of Duisburg-Essen, CAM-D Technologies GmbH, Schützenbahn 70, D-45117, Essen, Germany; 3 Institute for Molecular Enzyme Technology, Heinrich-Heine-University of Duesseldorf, Research Centre Juelich, Stetternicher Forst, D-52425, Juelich, Germany; 4 Current adress: Institute for Microbiology, Technische Universität Braunschweig, Spielmannstrasse 7, D-38106, Braunschweig, Germany; 5 Current adress: Institute for Pharmaceutical Biotechnology/ Ulm Centre for Peptide Pharmaceuticals, University Ulm, Albert-Einstein-Allee 11, D-89081, Ulm, Germany

**Keywords:** Biofilm, Lipase, Polysaccharide, Interaction, Enzyme stability, Molecular modeling

## Abstract

**Background:**

As an opportunistic human pathogen *Pseudomonas aeruginosa* is able to cause acute and chronic infections. The biofilm mode of life significantly contributes to the growth and persistence of *P*. *aeruginosa* during an infection process and mediates the pathogenicity of the bacterium. Within a biofilm mucoid strains of *P*. *aeruginosa* simultaneously produce and secrete several hydrolytic enzymes and the extracellular polysaccharide alginate. The focus of the current study was the interaction between extracellular lipase LipA and alginate, which may be physiologically relevant in biofilms of mucoid *P*. *aeruginosa*.

**Results:**

Fluorescence microscopy of mucoid *P*. *aeruginosa* biofilms were performed using fluorogenic lipase substrates. It showed a localization of the extracellular enzyme near the cells. A microtiter plate-based binding assay revealed that the polyanion alginate is able to bind LipA. A molecular modeling approach showed that this binding is structurally based on electrostatic interactions between negatively charged residues of alginate and positively charged amino acids of the protein localized opposite of the catalytic centre. Moreover, we showed that the presence of alginate protected the lipase activity by protection from heat inactivation and from degradation by the endogenous, extracellular protease elastase LasB. This effect was influenced by the chemical properties of the alginate molecules and was enhanced by the presence of O-acetyl groups in the alginate chain.

**Conclusion:**

We demonstrate that the extracellular lipase LipA from *P*. *aeruginosa* interacts with the polysaccharide alginate in the self-produced extracellular biofilm matrix of *P*. *aeruginosa* via electrostatic interactions suggesting a role of this interaction for enzyme immobilization and accumulation within biofilms. This represents a physiological advantage for the cells. Especially in the biofilm lifestyle, the enzyme is retained near the cell surface, with the catalytic centre exposed towards the substrate and is protected from denaturation and proteolytic degradation.

## Background

*Pseudomonas aeruginosa* is a Gram-negative bacterium which is ubiquitous in water and soil. It is able to produce and secrete several hydrolases which are important for nutrition of the bacterium, for biofilm structure [1] and, moreover, as virulence factors [2]. As an opportunistic human pathogen, *P*. *aeruginosa* can cause severe acute and chronic infections, especially in immuno-compromized patients. In addition to infections of the urinary tract, wounds, middle ear and eyes, *P*. *aeruginosa* is well known as the causative agent of chronic lung infections of cystic fibrosis (CF) patients [3]. Most of these infections are biofilm-associated [4,5]. Biofilms represent a bacterial state of life in which the cells are attached to biotic or abiotic surfaces or to each other. Thereby, they are embedded in a matrix of self-produced extracellular polymeric substances (EPS). Different amounts of polysaccharides, lipids, nucleic acids and proteins can be detected in the EPS matrix of biofilms formed by *P*. *aeruginosa*. Part of the proteins show enzyme activities *in vitro* and *in vivo*. The expression of several exoenzyme encoding genes was detected in the sputum of infected CF-patients by transcriptome analysis [6] and the presence of significant levels of extracellular enzyme specific antibodies in sera of infected CF patients is an indirect evidence for the production of extracellular enzymes during infection processes [7,8]. Therefore, the biofilm matrix of *P*. *aeruginosa* was described as a reservoir of enzymes [9].

The main extracellular enzymes produced by *P*. *aeruginosa* are type I and II-secreted hydrolases, including alkaline protease [10], elastase A (LasA) and B (LasB) [11], phospholipase C [12] and lipases [13,14]. These enzymes alone or synergistically with others are causing cell death, severe tissue damage and necrosis in the human host [2,15,16]. The simultaneous production of these exoenzymes and polysaccharides were described for *P*. *aeruginosa*[17,18]. During persistent CF-lung infections the conversion to a mucoid, i.e. an alginate overproducing phenotype is commonly observed [19]. Alginate is a high-molecular weight extracellular copolymer consisting the uronic acid monomers β-D-mannuronate and its C-5 epimer α-L-guluronate, which are linked by 1,4-glycosidic bonds [20,21]. These components are arranged in homopolymeric blocks of poly-β-D-mannuronate and heteropolymeric sequences with random distribution of mannuronate and guluronate residues [22]. In contrast to the alginate produced by brown algae the alginate from *P*. *aeruginosa* contains O-acetyl groups on the C2- and/or C3-position of the β-D-mannuronate residues. This acetylation significantly influences the physico-chemical properties of the polymer, such as the viscosity [23,24], the ability to bind divalent cations [23,25] and the water-binding capacity [26]. All of these features are important for the structure and the mechanical stability of the biofilm [24,27,28]. The extracellular alginate forms a highly hydrated matrix in which the bacteria cells are embedded. It can protect the cells from dehydration, the activity of antimicrobial substances as antibiotics [29] and disinfectants [30] and, moreover, protects the cells from the immune system during the infection process [31,32]. Several reports described the binding of extracellular enzymes such as lipases to this polysaccharide [33-35], but the type and molecular mechanism of this interaction are still unclear.

Lipases (EC 3.1.1.3) are physiologically and biotechnologically relevant enzymes. In addition to their natural function (hydrolysis of triglycerides), lipases are also able to recognize various substrates and catalyze regio- and enantioselective hydrolysis of many esters. The main extracellular lipase of *P*. *aeruginosa* is the 29 kDa lipase LipA [13], which belongs to the I.1 family of lipases [36]. X-ray studies showed that lipases of this family exhibit an α-helical lid structure, which closes the active centre of the enzyme [37]. The open, active conformation occurs only in contact with the substrate. This complex mechanism is called interfacial activation and can be mediated by a large range of hydrophobic substances, including lipopolysaccharides (LPS) [13]. However, LipA exhibits a lid structure, it does not show an interfacial activation, because interaction with hydrophobic outer membrane components let to a permanent open conformation [13,38]. Lipase LipA is transported across the cell envelope by the type II secretion system, the main two-step ATP-dependent process of Gram-negative bacteria [39]. It has been reported that mucoid *P*. *aeruginosa* strains showed up to 9-fold higher lipase activity than their spontaneous non-mucoid counterparts [40]. The exogenous supplementation of purified bacterial alginate from *P*. *aeruginosa* and *Azotobacter vinelandii* and also algal alginate to the culture media of non-mucoid *P*. *aeruginosa* strains increases the release of extracellular lipase from the bacterial cells [33]. It has been hypothesized that this enhanced release of lipase was due to a non-covalent association between lipases and alginate [33]. The co-secretion of LipA and alginate from *P*. *aeruginosa* cells may reinforce the synthesis of lipases. Thereby, the removal of the enzyme from the direct cell surface acts as a signal for the bacterial cell [41]. The interaction between lipase and alginate was further used for lipase purification strategies by ethanolic co-precipitation of the two molecules [34,35]. Beside the biotechnological relevance of this finding, the physiological function of the interaction between extracellular lipase and the polysaccharide alginate for the bacterium remains unclear.

Here, we demonstrate that lipase LipA from *P*. *aeruginosa* binds to the extracellular polysaccharide alginate by electrostatic interactions. This interaction localizes the enzyme near the cell surface and enhances the stability of the enzyme against heat and degradation by endogenous proteases.

## Results and discussion

### Expression of lipase in mucoid *Pseudomonas aeruginosa* biofilms

The activity of extracellular lipolytic enzymes in *P*. *aeruginosa* was investigated in biofilms grown on the surface of membrane filters placed on agar plates (PIA) at 36°C for 24 h (Table 1). Biofilms were grown from mucoid environmental strain *P*. *aeruginosa* SG81, strain SG81Δ*lipA* defective for LipA production, strain SG81Δ*lipA*::*lipA* with complementation of the *lipA* gene deletion *in trans* by plasmid pBBL7, LipA-overproducing strain SG81*lipA* + and vector control strain SG81MCS. The membrane filter biofilm model mirrored biofilms in environmental habitats as found in soil or on leaves and also biofilms involved in infections as for example lung infections of cystic fibrosis patients [42-44].

**Table 1:** **Cell density, unronic acid (alginate) content and extracellular lipase activity of agar-grown *P. aeruginosa *biofilms**

**Strain**	**Bacterial density × 10**^**9 **^**(cells/cm**^**2**^**)**	**Uronic acids (μg/cm**^**2**^**)**	**Lipase activity (nmol/min x cm**^**2**^**)**
SG81	1.4 ± 0.3	0.22 ± 0.01	0.12 ± 0.01
SG81MCS	1.3 ± 0.2	0.23 ± 0.01	0.14 ± 0.01
SG81Δ*lipA*	1.2 ± 0.1	0.22 ± 0.01	0.0 ± 0.0
SG81Δ*lipA*::*lipA*	1.5 ± 0.6	0.23 ± 0.03	6.50 ± 0.1
SG81*lipA*+	1.4 ± 0.2	0.23 ± 0.01	63.02 ± 5.2

The mucoid parent strain *P*. *aeruginosa* SG81 and its derivative strains (vector control SG81MCS, *lipA* mutant SG81Δ*lipA*, complementation strain SG81Δ*lipA*::*lipA* and *lipA* overexpression strain SG81*lipA*+) were tested. The results are expressed as mean values of four independent experiments.

The biofilms of the five strains revealed comparable cell densities between 1.2 and 1.5 × 10^9^ cells/cm^2^ (Table 1). Extracellular lipase activity was determined in cell-free supernatants of biofilm suspensions by a photometric assay, using para-nitrophenylpalmitate (pNPP) as a substrate. The parent strain and the vector control strain showed similar levels of extracellular lipase activity, whereas no extracellular lipase activity was detected in biofilms of the *lipA* mutant. Complementation of *lipA* in strain SG81Δ*lipA*::*lipA* restored lipase activity, and the *lipA* overexpression strain displayed significantly enhanced lipase activity that was 525-fold higher compared with the parent strain SG81 (Table 1). Uronic acids (alginate) were detected in all biofilms at nearly the same levels, indicating that alginate production was not influenced by the differential expression of lipase activities.

### Localization of lipase activity in *Pseudomonas aeruginosa* biofilms

**Figure 1:**
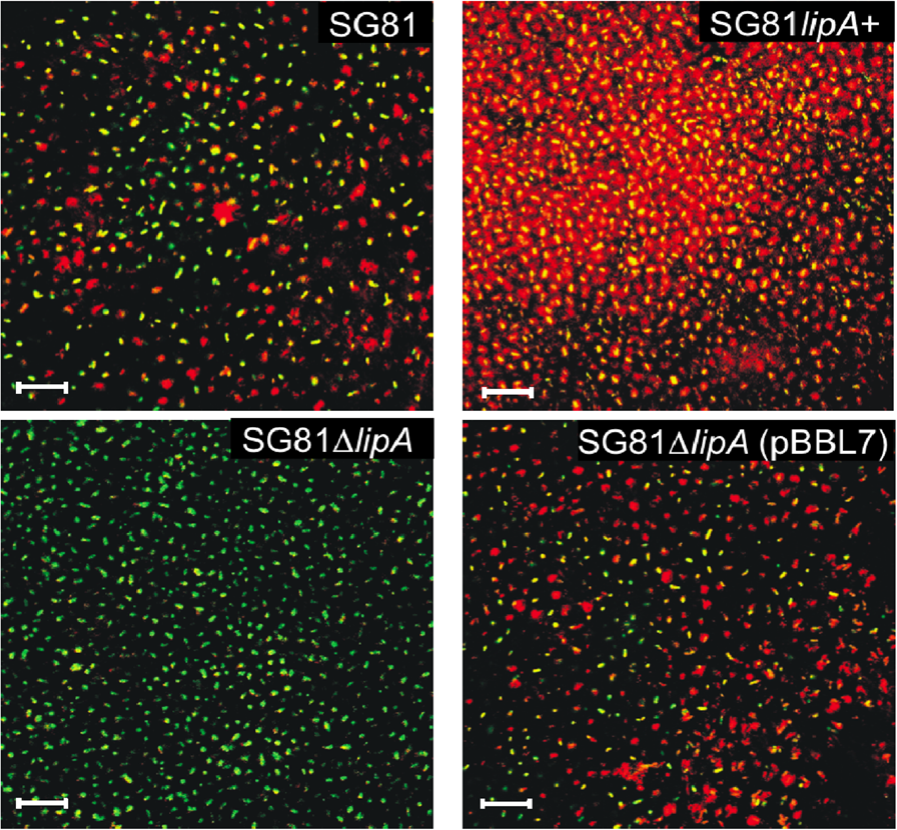
**Visualization of lipase activity in biofilms of *P. aeruginosa.*** Membrane filter biofilms (PIA + Ca^2+^, 24 h, 36°C) of the parent strain *P*. *aeruginosa* SG81, the *lipA* overexpression strain SG81*lipA*+, the *lipA* defect mutant SG81Δ*lipA* and their corresponding complementation strain SG81Δ*lipA*::*lipA* were stained using the lipase substrate ELF®-97-palmitate. Shown are CLSM micrographs (optical section in the vertical middle of the biofilms) at a 400-fold magnification. For cell staining SYTO 9 (green) were used. Lipase activity, red; cells, green; overlay, yellow. The bars indicate 20 μm.

In order to visualize lipase activity *in situ*, fluorescence micrographs of biofilms of the mucoid *P*. *aeruginosa* strain SG81 and its derivates were made using the fluorigenic lipase substrate ELF^®^-97-palmitate (Figure 1). An emulsion of the water insoluble ELF-97^®^-palmitate was prepared using sodium desoxycholate and gum arabic for emulsification and stabilisation of the substrate according to the well-established method for lipase activity determination with pNPP as a substrate [45]. Biofilms were grown on agar medium (PIA) supplemented with 0.1 M CaCl_2_ for stabilization of the biofilm matrix, since Ca^2+^ ions enhance the mechanical stability of *P*. *aeruginosa* biofilms by complexing the polyanion alginate [25,28,46]. This facilitates the treatment of the biofilms necessary for activity staining and subsequent observation by confocal laser scanning microscopy (CLSM).

A heterogeneous distribution of lipase activity within the biofilms was observed (Figure 1). Cellular activity in most of the cells indicated by the yellow colour and extracellular red-coloured regions surrounding the cells could be distinguished. Significantly more extracellular lipase activity was detected in the LipA overproducing strain *P*. *aeruginosa* SG81*lipA*+, indicating that the visualized extracellular lipase activity was mainly based on the activity of LipA. No extracellular but weak cell-associated activity was observed in the lipase mutant *P*. *aeruginosa* SG81Δ*lipA*. This can be explained by the activity of other lipolytic enzymes such as the outer-membrane bound esterase EstA, which is able to degrade palmitate [14,47]. The second extracellular lipase LipC of *P*. *aeruginosa* is unable to degrade palmitate ester substrates (personal communication). Furthermore, a deletion within the foldase gene *lipH* may also affect folding and activity of LipC [39]. The defect of extracellular lipolytic activity could be complemented by the expression of *lipA in trans* from the plasmid pBBL7. Accordingly, the complementation strain *P*. *aeruginosa* SG81Δ*lipA*::*lipA* revealed a level of lipase activity staining of the biofilms similar to the parent strain *P*. *aeruginosa* SG81. The biochemical detection of lipase activity in cell-free material from biofilms and the *in situ* visualization of lipase activity in the intercellular space of biofilms using palmitate-based enzyme substrates indicate that extracellular lipase is expressed in biofilms of mucoid *P*. *aeruginosa*, so that the biofilm matrix represents a reservoir of lipase molecules. By the use of strains that differ in their ability of producing LipA indicated that the major activity of extracellular lipase is due to the presence of lipase LipA.

### Binding of lipase LipA to alginate

Previous *in vitro* studies have demonstrated the stimulation of lipase release from non-mucoid wild-type *P*. *aeruginosa* by the addition of purified algal and bacterial alginate to cell suspensions. Moreover, the interaction of lipases and algal alginate with a concomitant stabilization of the enzyme against ethanol-induced denaturation was shown *in vitro*. On the basis of these observations we hypothesized that extracellular lipase in mucoid *P*. *aeruginosa* biofilms might be bound to the alginate in the EPS matrix. Therefore, *in vitro* binding studies in a microtiter plate assay were conducted using purified LipA from *P*. *aeruginosa* and bacterial alginate isolated from mucoid *P*. *aeruginosa* SG81 biofilms. For comparison, different neutral (dextran, levan) and negatively charged polysaccharides (algal alginate, xanthan) were tested. The immobilization of the polysaccharides on the polystyrene of the microtiter plate was verified by carbohydrate quantification. Binding of polysaccharides to wells of polystyrene microtiter plates in a concentration of 0.01 - 1.0 mg/ml was also shown before [48].

**Figure 2:**
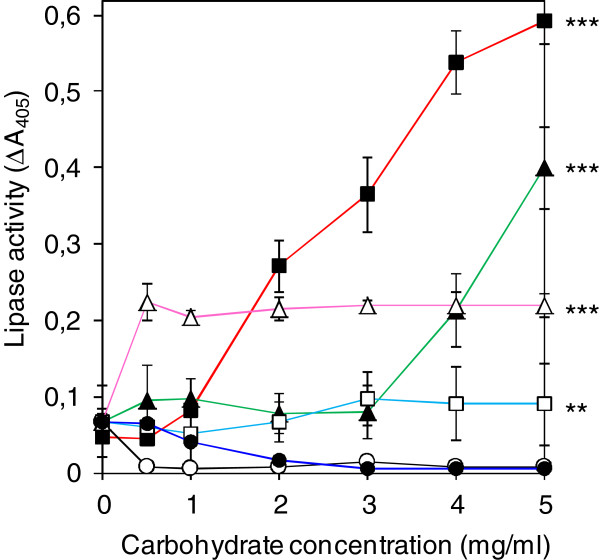
**Binding of purified lipase LipA from *P. aeruginosa *to polysaccharides.** Purified lipase LipA (36 ng/ml) from *P*. *aeruginosa* was incubated at 30°C in microtiter plates in the absence (−○-) and in the presence of immobilized polysaccharide films of (−■-) bacterial alginate from *P*. *aeruginosa* SG81 shown in red, (−▲-) xanthan shown in green, (−Δ-) algal alginate shown in pink, (−□-) levan shown in bright blue and (−●-) dextran shown in dark blue. Represented are carbohydrate concentrations used for coating of the microtiter plate wells. The bound lipase was detected by activity measurements using pNPP as substrate. Results are shown as mean of five independent experiments +/− standard deviations. Significance of differences in lipase binding between coated and uncoated wells was calculated by ANOVA for the highest tested polysaccharide concentration. *** *p* < 0.001; ** *p* < 0.01.

After two washing steps with 200 μl water each, a significantly increased lipase activity was detected in the wells of the microtiter plate with increasing amounts of bacterial alginate used for coating of the wells (Figure 2). This observation indicated that LipA bound to the immobilized bacterial alginate in a concentration-dependent manner. In the absence of polysaccharides no lipase activity was detected within the microtiter plate after the performed washing steps (∆A_405_ ≤ 0.07) indicating that LipA did not bind to the polystyrene surface (Figure 2). Without washing the lipase activity was ∆A_405_ = 0.8 +/− 0.1 independent of the presence or absence of polysaccharides. This result indicated that no interfacial activation of the lipase occurs by the presence of polysaccharides. It was reported that the enzyme exhibits a permanent open conformation [13]. Interestingly, no binding of LipA to the neutral polysaccharide dextran (poly-α-D-glucose) and only minor binding of LipA to levan (poly-β-D-fructose) was detected. These results suggested an influence of negative charges of the polysaccharides on the binding of lipase. A binding of LipA to xanthan was observed only at high concentrations of the polysaccharide. Xanthan is a heteropolymer of glucose, mannose and glucuronic acid, which is substituted with acetate and pyruvate residues. Therefore, this polysaccharide displays neutral as well as anionic properties and thus the charge density of xanthan was reduced in relation to alginate. Consequently, higher concentrations of this polysaccharide seemed to be necessary to bind measurable amounts of lipase molecules. In comparison to bacterial alginate, algal alginate showed a minor binding capaticity. However, binding of lipase to algal alginate was reported previously [34]. In contrast to bacterial alginate of *P*. *aeruginosa*, the algal alginate lacks O-acetyl groups and comprises a different monomer sequence which is characterized by the presence of guluronic acid rich regions (G-blocks) [22,49]. Since other studies did not reveal an influence of the O-acetyl groups on the binding of lipases [33] the here observed effect might be based on the different monomer structure of algal and bacterial alginates. It was shown that within the G-blocks of algal alginates specific intra- and intermolecular structures were formed (egg box). Within the egg boxes negative charges of the alginate molecules are directed to each other and are complexed via divalent cations. Thereby, the negative charges were shielded [50].

In summary, the experiments suggest that the binding of lipases to alginate depends on the negative charged monomers of the polysaccharide indicating ionic interactions between the molecules.

### Heat stabilization of lipases by polysaccharides

**Figure 3:**
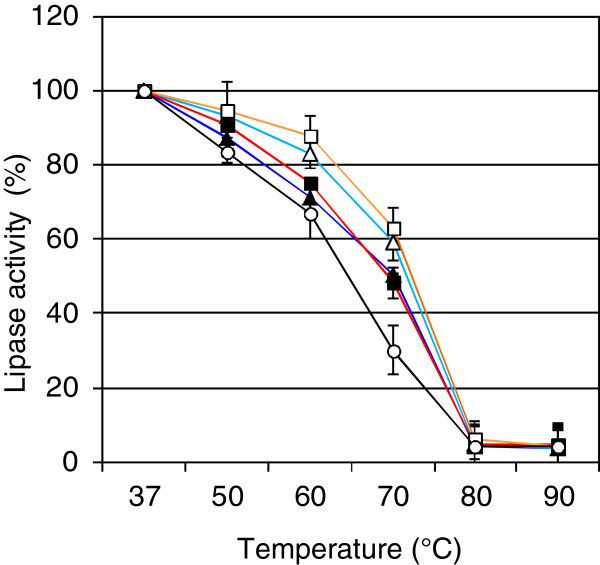
**Temperature-dependent heat inactivation of lipase LipA.** Purified lipase LipA (18 ng/ml) from *P*. *aeruginosa* was incubated for 20 min in the absence (−○-) and in the presence of 1 mg/ml (−■-) bacterial alginate from *P*. *aeruginosa* SG81 shown in red, (−**-**-) deacetylated bacterial alginate from *P*. *aeruginosa* SG81 shown in orange, (−**♦**-) bacterial alginate from *P*. *aeruginosa* FRD1 shown in dark blue, (−**◊**-) bacterial alginate from *P*. *aeruginosa* FRD1153 shown in bright blue. Results are shown as mean of three independent experiments with standard deviations.

To investigate the biological impact of the interaction between lipase and bacterial alginate, heat inactivation experiments were performed. Incubation of purified lipase for 20 min at different temperatures in the presence and absence of polysaccharides showed an obvious influence of alginate on the stability of the lipase activity (Table 2). Without heat treatment (37°C) lipase activity stayed constant over 20 min in the presence and absence of polysaccharides at ΔA_401_ = 0.66 +/− 0.15 corresponding to an activity of 2.3 +/− 0.5 nmol/min × μg protein. Furthermore, at temperatures up to 50°C the lipase seemed to be generally stable. The addition of polysaccharides only had a minor effect. At higher temperatures (> 80°C) the protective effect of polysaccharides was lost. However, at approximately 70°C a significantly increased temperature tolerance of the lipase was observed in the presence of bacterial alginate. Interestingly, a 2–3°C higher heat tolerance was obtained by the addition of alginates without O-acetyl groups compared to their O-acetylated equivalent (Figure 3). This effect was slightly stronger for the chemically deacetylated alginate from *P*. *aeruginosa* SG81 than for the alginate of the O-acetylation mutant *P*. *aeruginosa* FRD1153. This might be explained by the fact that the alginate of *P*. *aeruginosa* FRD1153 still contained a residual of 9% (w/w) of O-acetyl groups, whereas the chemically deacetylated alginate of *P*. *aeruginosa* SG81 was free of O-acetyl groups [24]. No protection of lipase activity was obtained by the addition of dextran and minor in the presence of algal alginates. Xanthan showed comparable protection ability as the bacterial alginate of *P*. *aeruginosa* SG81. These results were in accordance with the finding that the lipase did not or only slightly bind to these polysaccharides at a concentration of 1 mg/ml in the microtiter plate assay (Figure 2).

**Table 2:** Inactivation temperatures of lipase LipA calculated by extrapolation of the linear gradient of the heat inactivation curves

**Sample**	**T**_**100**_	**T**_**50**_	**T**_**0**_
	**(°C)**	**(°C)**	**(°C)**
Tris–HCl buffer (control)	45.0 +/− 2.5	63.8 +/− 1.1	82.7 +/− 2.9
Alginate FRD1	45.1 +/− 3.5	72.2 +/− 2.6	101.7 +/− 2.8
Alginate FRD1153	47.3 +/− 2.2	76.7 +/− 1.2	106.2 +/− 3.2
Alginate SG81	47.9 +/− 2.5	70.3 +/− 3.3	91.0 +/− 3.0
Alginate SG81, deacetylated	49.2 +/− 3.5	78.5 +/− 1.9	109.0 +/− 3.0
Algal alginate	54.0 +/− 4.7	68.1 +/− 2.7	87.2 +/− 3.4
Dextran	46.1 +/− 3.2	66.1 +/− 3.2	86.2 +/− 3.4
Xanthan	47.8 +/− 3.9	74.1 +/− 1.5	95.4 +/− 2.7

The lipase activity was detected after 20 min incubation at different temperatures in the presence (1 mg/ml) and absence of polysaccharides. Three independent experiments were performed in duplicates. Shown are T_0_ representing the temperature of complete inactivation of lipase activity, T_50_ which represents the temperature at which the lipase activity was reduced by half and T_100_ designated the maximum temperature where lipase activity remained unaffected within 20 min of incubation.

**Figure 4:**
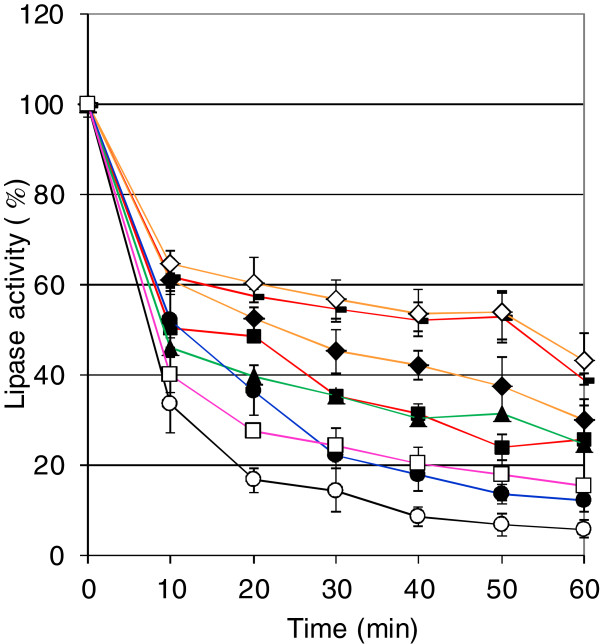
**Time-dependent heat inactivation of lipase LipA.** Purified lipase LipA (18 ng/ml) from *P*. *aeruginosa* was incubated at 70°C in the absence (−○-) and in the presence of 1 mg/ml (−■-) bacterial alginate from *P*. *aeruginosa* SG81 shown in red, (−**-**-) deacetylated bacterial alginate from *P*. *aeruginosa* SG81 shown in red, (−**♦**-) bacterial alginate from *P*. *aeruginosa* FRD1 shown in orange, (−**◊**-) bacterial alginate from *P*. *aeruginosa* FRD1153 shown in orange, (−□-) algal alginate shown in pink, (−▲-) xanthan shown in green and (−●-) dextran shown in blue. Results are shown as mean of five independent experiments with standard deviations.

In summary, the protection effect of alginate occurred mainly at temperatures between 50°C and 80°C. The inactivation of lipase activity at 70°C was investigated in more detail over an increased incubation time (Figure 4). In general, similar results were obtained even over a prolonged incubation time of 60 min. Bacterial alginates revealed a protection effect on LipA, which was found enhanced in case of deactetylated alginates. As seen above (Table 2), algal alginate did only slightly protect LipA from heat inactivation. Furthermore, dextran showed a protective effect on LipA activity at longer incubation times similar to that of algal alginate. This result was unexpected, since in contrast to algal alginate LipA did not bind to dextran in the microtiter plate assay. Interestingly, also over a prolonged time of incubation the addition of xanthan led to similar lipase activities as detected for bacterial alginate treated lipase. However, at the polysaccharide concentration of 1 mg/ml no binding of LipA was detectable (Figure 2). Nevertheless, this experiment indicated a comparable protective function of the negative-charged polysaccharides xanthan and bacterial alginate.

The interaction of enzymes with polysaccharides and the influence on the stability of the proteins was described earlier [35,51,52]. Heat stabilization effects were also reported for extracellular lipases from *P*. *aeruginosa*[34]. According to our results, the residual lipase activity after 60 min at 70°C in the presence of algal alginate was 15% of the initial activity. Also the stabilization of other bacterial extracellular enzymes by non-covalent associations with exopolysaccharides from the same bacterial species has been described before [53,54]. This thermostabilizing effect might be relevant for survival of biofilm grown *P*. *aeruginosa* cells in environmental habitats under conditions of elevated temperatures as for example sun-shined soil or heated water bodies.

### Protection of lipase from proteolytic degradation

**Figure 5:**
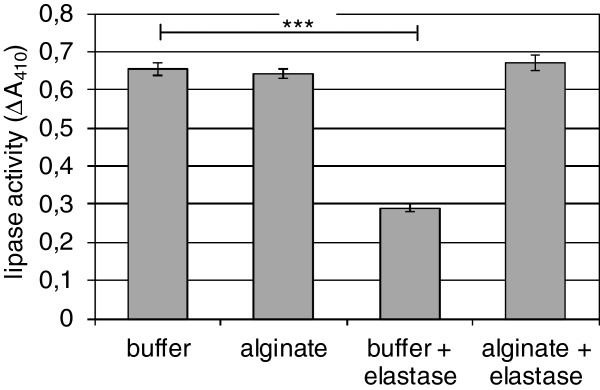
**Proteolytic degradation of lipase LipA through endogenous LasB.** Purified lipase LipA (18 ng/ml) from *P*. *aeruginosa* was incubated for 24 h at 37°C with 0.5 U purified LasB from *P*. *aeruginosa* (EMD4 Bioscience) in the absence and in the presence of bacterial alginate from *P*. *aeruginosa* SG81. A representative experiment of two independent experiments with standard deviations of the duplicates is shown. *** *p* < 0.001.

Another biological function of such interactions may be the stabilization of the enzyme and the protection from proteolytic degradation. To address this question, the stability of LipA in the presence of the endogenous elastase LasB purified from *P*. *aeruginosa* was tested (Figure 5).

A significantly decreased lipase activity was detected after 24 h incubation with LasB in the absence of alginate (*p* = 7.9 × 10^-6^). In contrast, no activity was lost in the presence of alginate. Moreover, the experiment again clearly showed that the addition of alginate did not stimulate the lipase activity, since the activity was similar in presence and absence of alginate. A stimulation of lipase activity would be a hint on conformational changes of the lipase protein. However, this seemed not to be happened. Lipase activity was found similar in the presence and in the absence of alginate without proteolytic treatment. Furthermore, no interfacial activation of the lipase was observed. This was expected as discussed above. However, elastase activity measured at the end of the experiment revealed constant over time. These results led to the suggestions that i) LasB is able to degrade the lipase LipA and ii) alginate protects the lipase molecule from degradation, possibly by covering of cleavage sites.

Elastase LasB has been described as one of the major extracellular proteases of *P*. *aeruginosa*[2]. The influence of LasB on the biofilm structure of mucoid *P*. *aeruginosa* was shown recently [1]. It was hypothesized that the proteolytic degradation of extracellular proteins mediated by LasB changes the physico-chemical properties of the EPS of *P*. *aeruginosa* and thereby, influences the structure of the biofilm [1]. Accordingly, a post-translational degradation of extracellular proteins during *P*. *aeruginosa* biofilm maturation was shown by proteome analysis [55]. Thereby, LasB has been identified as one of the enzymes involved [56]. Post-translational proteolytic processing cascades of extracellular proteins have also been found in other organisms [57,58].

### Modeling of interaction between lipase and polysaccharide alginate

**Figure 6:**
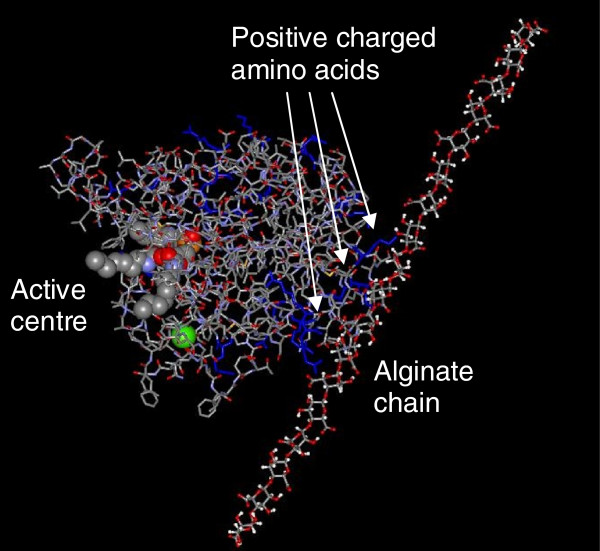
**Model of interaction between lipase A and alginate from *P*. *aeruginosa*.** Left: Lipase protein in presence of an inhibitor molecule in the active centre of the enzyme [37]. Furthermore, the co-factor molecule Ca^2+^ is indicated in green. Site chains of positively charged amino acids are shown in blue. Right: Section of an alginate molecule composed of negatively charged uronic acids in ball and stick representation. For better visibility the water in the reaction room is not shown (Redrawn from [9]).

Molecular modeling of inter- and intramolecular interactions between the extracellular lipase LipA and the exopolysaccharide alginate from *P*. *aeruginosa* was performed by molecular mechanics force field approach using a minimized energy simulation strategy (Figure 6). The crystal structure of the extracellular lipase LipA from *P*. *aeruginosa*[37] and a section of an alginate molecule were used. The modeling was carried out in presence and absence of water showing similar results. The calculations revealed that the interaction between lipase and alginate is mainly based on electrostatic interactions between negatively charged carboxyl groups of the polysaccharide and the positively charged amino acids of the protein as arginine, lysine and histidine (Figure 6, shown in blue). Mainly arginine, which is positively charged by the guanidinium group formed dominant interactions with the alginate chain. In accordance, the interaction remained stable even in the presence of water, whereas the histidine- and lysine-alginate interactions were slightly weakened. The binding to alginate let to a slight change in conformation of the whole protein, including the catalytic centre. However, it did not influence the activity of the enzyme (see above).

The interaction between alginate and lipases was hypothesized previously to be predominantly polar and non-specific, since addition of NaCl impaired co-precipitation, whereas Triton X-100 did not [34,41]. In a number of other studies the formation of complexes of alginate with various proteins such as trypsin, α-chymotrypsin, albumins, human leukocyte elastase and myoglobin has been demonstrated [41,59,60] underlining the non-specific binding of alginate to proteins.

Interestingly, the positively charged amino acids are localized on the surface of the protein mainly opposite of the active centre. This resulted in an immobilisation of the protein, while the reactive part of the biocatalyst remains unaffected and is directed to the surrounding environment and the substrate-containing reaction room.

## Conclusion

We demonstrate a binding of extracellular lipase LipA to the endogenous exopolysaccharide alginate from *P*. *aeruginosa* based on electrostatic interactions. This interaction has important biological advantages for the bacterium in biofilms. First, it prevents extracellular lipases from being rapidly diluted into the surrounding environment - the lipase accumulates and is immobilized near the cells within the alginate matrix, which facilitates the uptake of fatty acids released by the action of lipases. Moreover, the interaction between alginate and the backbone of the protein helps to direct the catalytic site of the enzyme to its substrate and therefore, can enhance the activity level.

A stabilization of the conformation of the enzyme by the interaction with the polysaccharide can be proposed. An evidence for this is the protection against proteolytic degradation and the enhanced heat tolerance of the enzyme. This gives an essential advantage for survival of *P*. *aeruginosa* under adverse environmental conditions.

## Methods

### Bacterial strains and cultivation

Bacterial strains and plasmids are listed in Table 3. The mucoid environmental strain *P*. *aeruginosa* strain SG81, the clinical strain FRD1 and its derivate FRD1153, which is defective in O-acetylation of the alginate [24,61,62] were used for the isolation of bacterial alginates. For production and isolation of the extracellular lipase LipA, *lipA* together with *lipH* encoding the corresponding chaperone LipH was homologous overproduced in *P*. *aeruginosa* PABST7.1/pUCPL6A [63]. For lipase activity visualization in biofilms the *lipA* defect mutant *P*. *aeruginosa* SG81Δ*lipA*, the corresponding complementation strain *P*. *aeruginosa* SG81Δ*lipA*::*lipA* and the *lipA* overexpression strain *P*. *aeruginosa* SG81*lipA* + carrying plasmid pBBL7 were used. This vector based on pBBR1MCS [64] and carries the genes *lipA* and *lipH* from *P*. *aeruginosa* PAO1 [1]. For construction of a Δ*lipA*-mutant from SG81 a Gm^r^ cassette was cloned into the suicide vector pMEΔAH11 [63] containing a 2.06 kbp *Kpn*I/*Xba*I-fragment with Δ(2/3 *lipA* 1/5 *lipH*). The resulting vector pMEΔAH::Ω-Gm^r^ was used for homologous recombination. All plasmids were transferred into *P*. *aeruginosa* SG81 via conjungation using *Escherichia coli* S-17.

**Table 3:** Bacterial strains and plasmids used in this study

**Strain/plasmids**	**Relevant genotype/ phenotype**	**Reference**
*E*. *coli S17*-*1*	*thi pro hsdR*^-^*M*^+^, chromosomally integrated [RP4-2 Tc::Mu:Km^r^::Tn7, Tra^+^ Tri^r^ Str^r^]	[65]
*P*. *aeruginosa*		[38]
PABST7.1/pUCPL6A	Overexpression of *lipA* and *lipH* from pUCPL6A	
FRD1	Mucoid Δ*mucA22* CF-lung isolate	[66]
FRD1153	Δ*algJ5*-mutant derived from FRD1, defect in O-acetylation of alginate	[61,62]
SG81	Mucoid biofilm isolate from technical water system	[67]
SG81MCS	Vector control pBBR1MCS	[1]
SG81Δ*lipA*	Δ(2/3 *lipA* 1/5 *lipH*)::Ω-Gm^r^ , deletion of *lipA* and *lipH*	This study
SG81Δ*lipA*::*lipA*	Deletion of *lipA* and *lipH* complemented *in trans* from pBBL7	This study
SG81*lipA*+	Expression of *lipA* and *lipH in trans* from pBBL7	[1]
pBBR1MCS	*lacZ*α Cm^r^ mob P_lac_, P_T7_	[64]
pBBL7	2.8 kbp *Xmn*I/*Sma*I fragment with *lipA*/*H* operon in pBBR1MCS under P_lac_ control	
pMEΔAH11	2.06 kbp *Kpn*I/*Xba*I-fragment with Δ(2/3 *lipA* 1/5 *lipH*) in pME3087	[63]
pMEΔAH::Ω-Gm^r^	1.6 kbp *Sma*I-fragment with Ω-Gm^r^ from pBSL142 in pMEΔAH11	This study

Biofilm cultures were grown for 24 h at 36°C on Pseudomonas Isolation Agar (PIA; Difco) in the form of confluent mucoid lawns. Cell numbers of biofilms, which were scraped from the agar surface and suspended in 0.14 M NaCl, were determined microscopically using a Thoma counting chamber. Cell-free EPS solutions prepared from the biofilm suspensions according to Tielen et al. [1] were used to measure uronic acid (alginate) concentration and lipase activity as described below. For CLSM analysis, biofilms were grown on membrane-filters (polycarbonate, size: 2.5 cm, pore size: 0.4 μm; Millipore, Billerica, Massachusetts) placed on PIA supplemented with 0.1 M CaCl_2_ for stabilization of the biofilm matrix as described previously [68].

### Visualization of lipase activity *in situ*

For visualization of lipase activity in biofilms of *P*. *aeruginosa* strains, ELF® 97 palmitate (Molecular Probes, Invitrogen GmbH, Karlsruhe, Germany) was used as a substrate. This enzyme substrate is cleaved by lipases to the water-insoluble ELF® 97 alcohol, which precipitates directly at the site of enzymatic hydrolysis, thus reporting the location of lipase enzyme activity, when visualized by fluorescence microscopy [69]. ELF® 97 palmitate obtained as a solid was dissolved at a concentration of 5 mM in isopropanol under weak heating. 1 ml substrate solution was mixed with 9 ml Sørensen phosphate buffer (pH 8.0) containing 20.7 mg sodium desoxycholate and 10 mg gum arabic. This substrate emulsion was stored in the dark for maximally 1 h.

24 h-old biofilms on membrane filters cultivated on calcium-amended PIA as described above were covered with 50 μl of the substrate emulsion. After incubation for 3 h at 30°C in the dark, lipase activities were detected by fluorescence microscopy using a LSM 510 confocal laser scanning microscope (Zeiss, Jena, Germany) with an excitation wavelength of 351 nm and emission long pass filter LP 505 nm or wide pass filter 505–550. In parallel, the biofilm cells were stained with SYTO 9 (Molecular Probes, Invitrogen GmbH, Karlsruhe, Germany) by adding 100 μl of SYTO 9 solution (1.5 μl SYTO added to 1 ml 0.9% (w/v) NaCl). After 15 min of incubation the fluorescence was recorded at an excitation wavelength of 488 nm by use of an argon laser in combination with an emission long pass filter LP 505 nm. Images were obtained with a Zeiss LD Achroplan 40x/0.60 NA objective. Digital image acquisition and analysis of the CLSM optical thin sections were performed with the Zeiss LSM software (version 3.2). For better visibility the fluorescence signals were stained with two different colors for imaging.

### Purification of extracellular lipase from *P*. *aeruginosa*

Lipase protein was purified by a two-step chromatographic procedure as described earlier [38]. In brief: lipase protein was produced in larger amounts by growing *P*. *aeruginosa* PABST7.1/pUCPL6A in 10 ml of double strength Luria Broth (2 × LB) containing 200 μg/ml carbenicillin and 50 μg/ml tetracycline in a 100 ml Erlenmeyer flask after inoculation with a single colony. Cells were grown overnight at 30°C, lipase gene expression was induced by addition of 0.4 mM IPTG and cells were further grown for 24 h.

Lipase expression cultures of recombinant *P*. *aeruginosa* were centrifuged; the culture supernatant was sterile filtered and concentrated by ultrafiltration by a factor of 15. One ml of the concentrated culture supernatant was mixed with 1 ml 10 mM Tris–HCl (pH 8.0), 100 mM NaCl and loaded onto a Fractogel EMD Bio SEC-chromatography column (length: 500 mm, inner diameter: 15 mm; Merck, Darmstadt, Germany) at room temperature. Proteins were eluted at 1 ml/min using the same buffer. Fractions containing the highest lipase activity (usually 15–20 fractions) were pooled and loaded onto an Uno-Q1 column (Bio-Rad, Munich, Germany), pre-equilibrated with buffer A (20 mM Tris–HCl pH 8.0, 100 mM NaCl) and connected to an FPLC unit (Pharmacia, Sweden). Proteins were eluted at 0.5 ml/min with the following NaCl gradient: 0–7 min with buffer A, 8–17 min from 100 mM to 400 mM NaCl in buffer A, 18–27 min from 400 mM to 1 M NaCl in buffer A, 28–32 min 1 M NaCl, 33–37 min from 1 M to 2 M NaCl in buffer A.

### Purification of bacterial alginate and other polysaccharides

Bacterial alginate was isolated and purified from biofilm cultures of *P*. *aeruginosa* SG81 (PIA, 36°C, 24 h) as described before [68]. Additionally, the bacterial polysaccharides dextran from *Leuconostoc mesenteroides* (Sigma-Aldrich, Munich, Germany), xanthan from *Xanthomonas campestris* (Sigma-Aldrich, Munich, Germany), levan from *Erwinia herbicola* (Fluka, Munich, Germany) and alginate (sodium salt) produced by brown algae (Manucol LHF, Nutra Sweet Kelco Company, Chicago, USA) were used. For further purification of dextran and algal alginate, 2 g of the polysaccharides were dissolved in 100 ml deionized water. After centrifugation of the solutions at 40,000 × g for 30 min the supernatants were collected, again centrifuged at 40,000 × g for 30 min and dialyzed (exclusion size: 12–14 kDa) twice against 5 l deionized water overnight. Finally, the polysaccharides were recovered by lyophilization. For further purification of xanthan and levan, the polysaccharides were dissolved in a concentration of 2.5 mg/ml in 50 mM Tris–HCl buffer (pH 7.5) containing 2 mM MgCl_2_. After addition of Benzonase (Merck, Darmstadt, Germany; final concentration 5 U/ml) and incubation for 4 h at 36°C, proteinase K (Sigma-Aldrich, Munich, Germany) was added (final concentration 5 μg/ml) followed by incubation at 36°C for 24 h. After centrifugation at 20,000 × g for 30 min, the supernatants were dialyzed (exclusion size: 12–14 kDa) twice against 5 l deionized water overnight and finally lyophilized.

### Chemical deacetylation of bacterial alginate

Deacetylation of bacterial alginates was performed as described before [20]. For complete deacetylation 25 mg purified alginate from *P*. *aeruginosa* SG81 was dissolved in 5 ml deionized water. After addition of 2.5 ml 0.3 M NaOH and incubation for 1 h at room temperature the pH was adjusted to 8.0 with 0.5 M HCl. Finally, the solution was dialyzed (exclusion size: 12–14 kDa) twice against 5 l deionized water overnight and lyophilized.

### Quantification of lipase activity

Lipase activity was measured with para-nitrophenyl palmitate (pNPP) as a substrate as described before [45]. An absorbance at 410 nm of 1.0 per 15 min corresponds to a lipase activity of 48.3 nmol/min x ml solution.

### Quantification of polysaccharides

Total carbohydrate and uronic acid (alginate) concentrations were determined with the phenol-sulfuric acid method [70] and the hydroxydiphenyl assay [71], respectively, using purified alginate from *P*. *aeruginosa* SG81 as a standard.

### Interaction of lipase with polysaccharides

For the investigation of interactions between lipase and polysaccharides a microtiter plate (polystyrene, Nalgene Nunc, Roskilde, Denmark) binding assay was applied. Purified polysaccharides were dissolved in 0.9% (w/v) NaCl solution and incubated for 15 min at 90°C to inactivate possibly remained enzymes. After cooling on ice, 100 μl of these polysaccharides in various concentrations (0–5 mg/ml) were immobilized by drying in the wells of microtiter plates at 30°C overnight, followed by a washing step with 200 μl distilled water (A. dest.) to remove NaCl. Afterwards 20 μl of purified lipase LipA from *P*. *aeruginosa* PABST7.1/pUCPL6A (72 ng/ml A. dest.) was added and incubated at 30°C for 30 min. Non-bound lipase was removed by two washing steps with 100 μl A. dest. each. Bounded lipase was detected via activity measurement in the microtiter plate using pNPP as substrate. The cleavage of the substrate was monitored at 405 nm in a microtiter plate reader. All experiments were performed in duplicates and repeated three times.

### Heat treatment of lipase

The stabilization of lipases through the interaction with alginate was investigated by heat treatment of purified lipase in presence and absence of polysaccharides. One volume purified lipase LipA from *P*. *aeruginosa* PABST7.1/pUCPL6A (36 μg/ml A. dest.) was mixed with one volume purified polysaccharides (2 mg/ml in 100 mM Tris–HCl buffer, pH 7.5), which were previously heated (15 min, 90°C) and afterwards cooled on ice to room temperature. After pre-incubation for 30 min at room temperature the samples were incubated for 0–60 min at 70°C to determine lipase inactivation kinetics. Moreover, the samples were incubated for 20 min at different temperatures (37°C; 50°C; 60°C; 70°C; 80°C; 90°C) to determine T_50._ T_50_ represents the temperature at which incubation for 20 minutes reduces the enzymes activity by half. Every 10 min the residual lipase activities were detected after cooling on ice, using pNPP as substrate. All experiments were performed in duplicates and repeated three times.

### Degradation of lipase by proteases

The protection of lipase from proteolytic degradation through the interaction with alginate was studied by using purified elastase LasB from *P*. *aeruginosa* (EMD4Biosciences, San Diego, USA). Briefly, 0.5 ml purified lipase LipA from *P*. *aeruginosa* PABST7.1/pUCPL6A (36 μg/ml A. dest.) was mixed with 0.5 ml purified polysaccharides (2 mg/ml in 100 mM Tris–HCl buffer, pH 7.5) previously heated for 15 min and 90°C and afterwards cooled on ice to room temperature. After pre-incubation for 30 min at room temperature, 20 μl purified elastase (0.1 mg/ml with 25 U/ml in A. dest.) were added. After 24 h incubation at 37°C the residual lipase activity was detected, using pNPP as a substrate. All experiments were performed in duplicates and repeated three times.

### Modeling of lipase-alginate interaction

The protein structure was based on the crystal structure of the lipase protein resulting from the X-ray diffraction analysis of a lipase protein [37]. The crystal structure was obtained from the RCSB protein data bank [72]. The hydrogens of the amino acids were adjusted according to the pKs values of the amino acids at a pH value of 7.0. Therefore, the resulting net charge of the protein was in accordance to an aqueous solution of pH = 7.0. Inter- and intramolecular interactions were calculated by a molecular mechanics force field approach. The here used atom types and atomic partial charges of the CHARMM27 force field were described before [73].

In order to calculate the lipase – alginate interaction a modified docking procedure was applied including a water shell around the protein and alginate chain. In this procedure the lipase and alginate atoms were randomly moved. This resulted in a slight rotation and translation of the molecules. In consequence the potential energy of the resulting structure was minimized and saved. The step of changing the atomic positions was repeated several thousand times and the potential energy differences between all collected structures were checked. The structures with the lowest potential energies were extracted.

### Statistical analyses

The significance of the data were analysed using the two-sample t-test and one way analyses of variance (ANOVA; [74]). A significant difference was considered to be *p* < 0.05.

## Competing interests

The authors declare that they have no competing interests.

## Authors’ contributions

PT performed the experiments, HK performed molecular modeling, JW conceived the study; PT, FR and JW wrote the manuscript. KEJ and HCF coordinate the work. All authors read and approved the final manuscript.
